# Factors associated with physical function capacity in an urban cohort of people living with the human immunodeficiency virus in South Africa

**DOI:** 10.4102/sajp.v75i1.1323

**Published:** 2019-09-09

**Authors:** Ronel Roos, Hellen Myezwa, Heleen van Aswegen

**Affiliations:** 1 Department of Physiotherapy, University of the Witwatersrand, Johannesburg, South Africa

**Keywords:** physical function capacity, HIV, 6-min walk test, pulmonary tuberculosis, exercise tolerance, reference equation

## Abstract

**Background:**

Effective disease management for people living with human immunodeficiency virus (PLWH) includes the encouragement of physical activity. Physical function capacity in PLWH may be influenced by a variety of factors.

**Objectives:**

This study describes the physical function capacity as assessed with the 6-minute walk test (6MWT) of an urban cohort of PLWH and determined whether a history of pulmonary tuberculosis (PTB), anthropometric measures, age and gender predicted distance walked.

**Method:**

Secondary data collected from 84 PLWH on antiretroviral therapy were analysed. Information included 6MWT distance, anthropometric measurements and demographic profiles. Descriptive and inferential statistics were undertaken on the data. A regression analysis determined predictive factors for 6MWT distance achieved. Significance was set at a *p*-value of ≤ 0.05.

**Results:**

The study consisted of 66 (78.6%) women and 18 (21.4%) men with a mean age of 39.1 (± 9.2) years. The 6MWT distance of the cohort was 544.3 (± 64.4) m with men walking further (602.8 [± 58.6] m) than women (528.3 [± 56.4] m); however, women experienced greater effort. The majority of the sample did not report a history of PTB (*n* = 67; 79.8%). Age, gender and anthropometric measures were associated with 6MWT distance, but of low to moderate strength. The regression equation generated included age and gender. This model was statistically significant (*p* < 0.00) and accounted for 34% of the total variance observed.

**Conclusion:**

Age and gender were predictive factors of physical function capacity and women experienced greater effort.

**Clinical implications:**

This study provides information on the physical function capacity of PLWH and a suggested 6MWT reference equation for PLWH in South Africa.

## Introduction

People living with the human immunodeficiency virus (PLWH) are encouraged to engage in physical activity and exercise to improve their health as it forms part of effective disease management. Moderate-intensity aerobic exercise is suggested as a safe method of improving cardiorespiratory function, muscle strength, body composition, depression and health-related quality of life (QOL) (O’Brien et al. 2016). Before individuals participate in an exercise programme, assessment of their physical function capacity is advised to determine how they respond to increased effort in a controlled environment (American College of Sport Medicine [ACSM] 2010b).

The 6-minute walk test (6MWT) is a sub-maximal exercise test that evaluates individuals’ physical function capacity and is used in a variety of clinical populations (Bohannon & Crouch 2017). During this test, the distance that an individual can walk for 6 min is determined (American Thoracic Society [ATS] 2002). The distance achieved during the 6MWT in PLWH often varies and may be less than that achieved by non-infected individuals (Mbada et al. 2013; Oursler et al. 2006). Oursler et al. (2006) noted this difference to be 8% as evaluated in men aged 40–69 years. In comparison, Beans et al. (2013) found no difference in 6MWT findings of men who were HIV positive compared to men who were HIV negative when participants had similar comorbidities and demographic profiles. Factors known to influence 6MWT distance walked in PLWH include inspiratory muscle weakness (Jerônimo et al. 2015); older age, current smoking status airflow limitations (Campo et al. 2014); peak oxygen utilisation (VO_2_ peak) (Beans et al. 2013; Oliveira et al. 2018; Oursler et al. 2009) and active pulmonary tuberculosis (PTB) co-infection (Pontororing et al. 2010).

Tuberculosis (TB) is an opportunistic infection frequently diagnosed in PLWH in resource-limited continents, such as Africa and Asia (Manosuthi, Wiboonchutikul & Sungkanuparph 2016). Despite the advances in the detection and management of TB, it remains the leading cause of death among PLWH (UNAIDS 2017). As a means of reducing TB-related deaths, high prevalence countries, such as South Africa, have adopted the 90–90–90 (diagnosed and sustained therapy and undetectable viral loads) treatment strategy for HIV and TB with 75% treatment for multidrug-resistant TB highlighted (South African National AIDS Council 2017). This treatment approach is set out in South Africa’s National Strategic Plan for HIV, TB and sexually transmitted infections (STIs) 2017–2022 (South African National AIDS Council 2017). Most research related to TB has focused on highlighting the burden of the disease and refining strategies to improve early detection, treatment and care as a means of lessening mortality. Few researchers have evaluated the long-term impact of this condition on individuals’ physical function and QOL. It is interesting that a plan to extend the 90–90–90 strategy to accommodate morbidity has been proposed, therefore suggesting a 90–90–90–100 strategy where 100% of services link chronic care to rehabilitation in PLWH (Hanass-Hancock et al. 2016).

Tuberculosis can significantly reduce individuals’ QOL (Brown et al. 2015; Guo, Marra & Marra 2009), but QOL can improve over time with successful TB management (Guo et al. 2009; Mthiyane et al. 2016). An issue that may contribute to the reduction in QOL is the effect that PTB has on individuals’ lung function. Active PTB and a history of PTB are known to reduce individuals’ lung function (Cole et al. 2016; Gupte et al. 2017). An important finding by Gupte et al. (2017) was that the reduction in lung function can progressively occur over time even when individuals with PTB have completed treatment.

The study conducted by Gupte et al. (2017) included PLWH with and without PTB. When the authors conducted the data analysis, they did a sub-analysis to further review how PTB influenced lung function outcomes over time. The suggested progressive changes in the lung function of PLWH could significantly impact the lung health of such individuals and possibly result in disability.

It is not known whether a history of PTB influences physical function capacity (measured using 6MWT distance) in individuals’ living with HIV. Considering the high burden of HIV and TB co-infection in sub-Saharan Africa and the fast track treatment strategies proposed, it seems prudent to evaluate this as related to physical function. Thus, the aims of our study were to describe the 6MWT findings of an urban cohort of South African PLWH, to determine whether a history of PTB, anthropometric parameters, age and gender were predictive factors of 6MWT distance achieved and to suggest a 6MWT reference equation for PLWH in Africa.

## Methods

### Study design and population

A secondary analysis of baseline data of a single-blind randomised control trial (RCT) of an urban cohort of PLWH on antiretroviral therapy for longer than 6 months who participated in a physical activity programme (Roos et al. 2014) provides the information for this study. Data collection for the RCT took place from June 2012 to August 2013.

Information from a systematic review performed by Ebrahim and Smith (1998) regarding the estimated change in systolic blood pressure (SBP) that occurs during an exercise programme was used as a guide for the sample size calculation. If the change in SBP is 6 mmHg (± 7) with alpha set at 5% and the power at 80%, then the sample calculated using a sample size calculator was estimated at 22 participants for the intervention, and 22 participants acting as controls. To account for loss to follow-up, a challenge faced by roll-out highly active antiretroviral therapy (HAART) centres in South Africa, and non-adherence to exercise reported in PLWH, 20% non-compliance and 20% drop-out rates, respectively, were selected as precautions for the eventuality of a decreasing sample size as the study progressed. This resulted in the sample used in the RCT study: 42 control and 42 intervention participants (Roos et al. 2014).

Individuals were recruited for the physical activity programme if they presented with risk factors of coronary heart disease as determined during a cross sectional study. Participants were consecutively sampled according to the following inclusion criteria: on HAART for 6 months or more, aged 20–65 years and ambulatory without an assistive device and willing to accept randomised group allocation. In addition, participants presented with one or more of the following: a pedometer physical activity level of less than 10 000 steps per day; a body mass index (BMI) value of ≥ 25 kg/m^2^; increased waist circumference (WC) (women ≥ 88 cm and men ≥ 102 cm); increased waist:hip ratio (women ≥ 0.85 and men ≥ 0.95); blood pressure values in the high normal range (SBP 130–139 mmHg and diastolic blood pressure [DBP] 85–89 mmHg) or mild hypertension range (SBP 140–159 mmHg and DBP 90–99 mmHg); and a known medical history of diabetes or hypertension or dyslipidaemia (and on medical management) (Roos et al. 2014). Individuals were excluded from participation in the study if they had a resting heart rate above 100 bpm or a blood pressure ≥ 160/100 mmHg; had any condition that was life threatening or could be aggravated by exercise; had an acute infection or active AIDS-defining opportunistic illness; were pregnant or had a newly documented mental illness. They attended an HIV clinic in Johannesburg.

### Data collection

All assessments were conducted by a research assistant in a private room except for the procedure related to the 6MWT which was carried out in a quiet corridor. The research assistant received training regarding the procedures to be conducted.

*Demographic profile*: Participants completed a demographic questionnaire. Their latest CD_4_ count and viral load values were collected from the clinic laboratory database. Self-reported history of PTB was collected from each participant during their initial interview when determining past medical history. Participants were asked if they currently had PTB or had PTB in the past and whether they received treatment for this condition. Participants’ resting vital signs (heart rate, respiratory rate and blood pressure) were assessed following the demographic profile review to ensure all participants were stable before physical function capacity assessment.

*Anthropometric measurements:* Participants were instructed to remove their shoes and any objects out of their pockets. They were weighed twice on a portable electronic scale. Prior to each measurement, the device was calibrated as per the manufacturer’s instructions. Weight measurements to the nearest 0.1 kg were taken. Participants were instructed to stand upright on a Micro Health stadiometer facing the research assistant with their head in a neutral position for height measurement. Height was measured twice to the nearest 0.1 cm. Participants’ BMI was calculated using the equation: BMI = weight (kg) ÷ height (m)² (ACSM 2010a). Waist circumference was measured using a non-stretch tape measure at the narrowest circumference halfway between the lowest rib and iliac crests (a horizontal measure was taken at the narrowest part of the torso: above the umbilicus but below the xiphoid process). Participants were upright, encouraged to exhale gently at the time of measurement with the arms at their sides, feet together and a relaxed abdomen (ACSM 2010). Two measurements were taken.

Participants’ hip circumference was measured at the broadest circumference of the participants’ buttocks.

For each anthropometric measurement assessed, the mean of the two values taken was determined and recorded for data analysis.

*Physical function capacity:* Participants’ physical function capacity was determined with the 6MWT. A 30-m distance was demarcated with two cones in a quiet hospital corridor. Each participant sat on a stool for a minimum of 5 min prior to the start of the 6MWT to rest before vital signs were measured. Pulse rate and oxygen saturation were measured with a Nonin Onyx^®^ 119 550 finger probe pulse oximeter. After these two measurements, participants’ respiratory rate was evaluated. Level of breathlessness and fatigue according to the Borg scale was assessed next. The recommendations suggested by the ATS guidelines (2002) on how to use the Borg scale when assessing breathlessness and fatigue were followed. The participants were shown the scale and asked: ‘Please grade your level of shortness of breath and breathlessness using this scale’. After feedback received on their breathlessness level, the following was asked: ‘Please grade your level of fatigue and tiredness using this scale’. At the completion of the exercise test, participants were reminded of their pre-test grading of breathlessness and fatigue before they re-assessed their post-test level of symptoms as per the pre-test procedure. If participants had difficulty understanding explanations, an interpreter assisted with translation. The walking procedure was demonstrated and participants were instructed to walk as many times as possible for 6 min between the two cones. They were informed that they could stop at any time if they felt unwell. The test was performed according to the standardised prompts as per the ATS guidelines (ATS 2002). All pre-test parameters were repeated at the completion of the test. Participants’ actual distance walked was compared to their predicted distance walked using the equation: expected 6MWT distance = 868.8 – (2.99 × age) – (74.7 × gender) (men = 0; women = 1) (Gibbons et al. 2001). The 6MWT work was calculated for the study participants.

The 6MWT work is a product of body weight in kilograms times distance walked in metres, as body weight directly affects the work, or energy required to perform the walk (Carter et al. 2003; Chuang, Lin & Wasserman 2001). The authors suggest including this calculation when reviewing findings from the 6MWT to enhance the understanding of functional capacity (Carter et al. 2003; Chuang et al. 2001).

### Data analysis

Data analysis was performed using IBM SPSS 24 (IBM Corp. 2016). The Shapiro–Wilk test and histograms were used to test for the normality of data distribution. Demographic information, resting vital signs, anthropometric parameters and 6MWT findings are presented as means ± standard deviation (SD) or median interquartile ranges. Differences between groupings were assessed using an independent *t*-test or Mann–Whitney *U* test. Associations between the 6MWT distance and parameters assessed were established with the Pearson and Spearman correlation coefficients. Stepwise (forward) multiple regression analysis (with *F* probability to enter ≤ 0.05 and probability to remove ≥ 0.1) was used to determine the predictor variables for the 6MWT. The following variables were included in the stepwise analysis: age, gender, height, weight, waist and hip circumference and a history of PTB. A *p*-value of ≤ 0.05 indicates significance.

## Results

### Characteristics of study participants

The sample consisted of 66 (78.6%) women and 18 (21.4%) men (Table 1). The CD_4_ count, BMI and hip circumference were significantly higher in women compared to men, but men were significantly taller than women (Table 1). Seventeen (20.2%) participants reported a history of PTB and completed treatment for this condition. No participant had active PTB at the time of assessment. Nine (10.7%) participants were current smokers (*n* = 3 PTB group; *n* = 6 non-PTB group), nine (10.7%) participants had known hypertension and one (1.25%) participant had known diabetes mellitus, and in all cases pharmacological treatment was provided for these chronic conditions. The mean time since knowing their HIV status was 5.1 (± 2.6) years and the years when antiretroviral therapy was initiated in study participants were: 2009 (*n* = 4; 4.8%); 2010 (*n* = 30; 35.7%); 2011 (*n* = 49; 58.3%); and 2012 (*n* = 1; 1.2%).

**TABLE 1:** Characteristics of study participants (*n* = 84).

Variable	Total (*n* = 84)			Women (*n* = 66)			Men (*n* = 18)			*p*
	Mean	Standard deviation	Median interquartile ranges	Mean	Standard deviation	Median interquartile ranges	Mean	Standard deviation	Median interquartile ranges	
**Age, years**	39.1	± 9.2	-	38.5	± 8.9	-	41.2	± 10.2	-	0.27
**Immune parameters**										
CD_4_ count, cells/mm^3^	331.0	-	212.8–438.8	371.5	-	247.3–486.3	227.5	-	140.0–307.5	0.00
Viral load, copies/mL	41.0	-	0.0–296.3	40.0	-	0.0–263.5	71.5	-	30.0–319.8	0.42
**Anthropometric parameters**										
Height, cm	165.5	± 7.1	-	163.3	± 5.3	173.5	± 7.1	-	0.00
Weight, kg	69.0	-	60.3–75.4	69.7	-	60.1–76.1	66.2	-	60.3–73.3	0.37
BMI, kg/m^2^	24.5	-	21.6–28.2	26.2	-	22.2–29.5	22.0	-	20.2–24.2	0.00
WC, cm	79.5	-	73.3–86.9	79.5	-	74.4–87.9	79.6	-	69.8–83.3	0.30
Hip circumference, cm	102.5	-	93.5–111.0	104.1	-	96.4–113.3	95.6	-	88.9–99.0	0.00
**Vital signs**										
Resting RR, breaths per minute	14.5	± 2.2	-	14.6	± 2.2	-	14.1	± 2.2	-	0.35
Resting HR, bpm	78.9	± 8.3	-	79.1	± 10.7	-	78.6	± 13.4	-	0.87
Resting SBP, mmHg	120.7	± 11.7	-	120.5	± 11.7	-	121.5	± 11.9	-	0.75
Resting DBP pressure, mmHg	78.8	± 8.3	-	78.4	± 8.4	-	80.0	± 8.1	-	0.47

BMI, body mass index; bpm, beats per minute; cells/mm^3^, cells per cubic millimetre; cm, centimetre; copies/mL, copies per millilitre; DBP, diastolic blood pressure; HR, heart rate; kg, kilogram; kg/m^2^, kilogram per square metre; mmHg, millimetre of mercury; RR, respiratory rate; SBP, systolic blood pressure; WC, waist circumference.

Findings are presented as means ± standard deviation (SD) or median interquartile ranges.

### Six-minute walk test findings

All participants (*n* = 84) were able to complete the 6MWT and none stopped for a rest because of breathlessness while performing the 6MWT. Eight (9.5%) participants reported other symptoms on the completion of the test, which include dizziness (*n* = 3; 3.6%), discomfort in the calves (*n* = 2; 2.3%), stiffness in the legs (*n* = 1; 1.2%), tingling in the thighs (*n* = 1; 1.2%) and feeling tired (*n* = 1; 1.2%).

Men walked significantly farther than the women during the 6MWT and achieved a higher percentage predicted value (Table 2). Women had a significantly higher heart rate and breathlessness level after the 6MWT compared to the men, and they also achieved a higher percentage of their age-predicted maximum heart rate (Table 2).

**TABLE 2:** The 6-min walk test findings of study participants (*n* = 84).

Variable	Total (*n* = 84)			Women (*n* = 66)			Men (*n* = 18)			*p*
	Mean	Standard deviation	Median interquartile ranges	Mean	Standard deviation	Median interquartile ranges	Mean	Standard deviation	Median interquartile ranges	
6MWT distance, m	543.8	-	509.0–577.8	538.6	-	497.3–567.0	595.2	-	564.9–627.8	0.00
6MWT percentage predicted achieved	78.2	± 8.2	-	77.6	± 8.0	-	80.1	± 8.7	-	0.00
6MWT work, kg/m	38 098.0	± 8 109.6	-	37 638.1	± 8620.2	-	39 784.4	± 5756.7	-	0.32
Pre-heart rate, bpm	76.9	± 14.0	-	77.8	± 13.4	-	73.7	± 15.9	-	0.27
Post-heart rate, bpm	104.8	± 22.5	-	107.7	± 21.2	-	94.1	± 24.6	-	0.02
HR_max_	180.9	± 9.2	-	181.5	± 8.9	-	178.8	± 10.2	-	0.27
Percentage of HR_max_ achieved	58.0	± 12.2	-	59.4	± 11.8	-	52.4	± 12.7	-	0.03
Pre-respiratory rate, breaths per minute	18.3	± 4.1	-	18.7	± 4.1	-	16.6	± 3.3	-	0.04
Post-respiratory rate, breaths per minute	24.5	± 5.4	-	25.1	± 5.7	-	22.6	± 3.8	-	0.08
Pre-oxygen saturation, %	96.7	± 2.7	-	96.7	± 2.9	-	96.7	± 1.8	-	0.98
Post-oxygen saturation, %	96.4	± 3.3	-	96.4	± 3.4	-	96.3	± 3.3	-	0.91
Pre-breathlessness level	0.2	± 0.7	-	0.3	± 0.8	-	0.0	± 0.0	-	0.19
Post-breathlessness level	1.0	± 1.7	-	1.2	± 1.9	-	0.2	± 0.4	-	0.04
Pre-fatigue level	0.2	± 0.6	-	0.2	± 0.7	-	0.0	± 0.1	-	0.26
Post-fatigue level	0.9	± 1.8	-	1.0	± 1.9	-	0.4	± 0.9	-	0.22

HRmax, maximum heart rate; m, metre; bpm, beats per minute; kg/m, kilogram per metre.

Findings are presented as means ± standard deviation (SD) or median interquartile ranges.

### Associations between parameters and 6-minute walk test distance

The associations between the parameters assessed and the 6MWT distance were as follows: age (*r* = −0.27, *p* = 0.00); gender (*r* = −0.49, *p* = 0.01); height (*r* = 0.31, *p* = 0.00); weight (*r* = −0.10, *p* = 0.46); BMI (*r* = −0.29, *p* = 0.01); WC (*r* = −0.21, *p* = 0.05); hip circumference (*r* = −0.21, *p* = 0.05) and history of PTB (*r* = 0.11, *p* = 0.31).

### Stepwise multiple regression analysis

The regression equation generated with the stepwise multiple regression analysis included age and gender. This model was statistically significant (*p* = 0.00) and accounted for 34% of the total variance of the 6MWT distance findings (Table 3). The equation for the regression line reflecting the predictive 6MWT distance for this cohort is: *y* = 699.50–80.95 (gender: women = 1; men = 0) – 2.35 (age).

**TABLE 3:** Stepwise regression results.

Model	Coefficient	Standard error	*p*	95% CI	Partial correlation
*r*^2^ = 0.34	-	-	-	-	-
Constant	699.50	29.04	0.000	641.71, 757.29	-
Gender (1 = women, 0 = men)	−80.95	14.19	0.000	−109.19, -52.72	−0.54
Age	−2.35	0.64	0.000	−3.61, -1.08	−0.38

CI, confidence interval.

## Discussion

Our study demonstrates that the presence of a past medical history of PTB was not associated with or predictive of participants’ 6MWT distance in this study population. It is possible that study participants had PTB at different time points and time since having been diagnosed and successful treatment for PTB would have influenced the extent to which they participated. It is known that a progressive decline in lung function may occur over time in individuals following successful treatment of PTB. It is for this reason that we decided to test the effect of a PTB history on physical function in PLWH. Less than a quarter of the cohort presented with a history of PTB and this may account for the fact that PTB did not seem to affect physical function capacity. A past medical history of PTB can reduce individuals’ lung function and lead to obstructive lung disease changes (Cole et al. 2016; Gupte et al. 2017; Jung et al. 2015). Gupte et al. (2017) noted the lung function decline to be a yearly loss of 35 mL (95% CI 2–68, *p* = 0.03) in Forced Expiratory Volume in 1 second (FEV_1_) and 57 mL (95% confidence interval [CI] 19–96, *p* = 0.003) in forced vital capacity (FVC) when compared to PLWH who did not have a history of previous PTB. A limitation of our study is that pulmonary function tests were not performed as part of the assessment of participants in the original study.

In addition to changes in lung function, Jung et al. (2015) reported that individuals with prior PTB were more likely to complain of respiratory symptoms, such as cough and shortness of breath. These symptoms influenced their QOL when compared to individuals without such a history. Respiratory symptoms, for example complaints of chronic cough, may also influence individuals’ physical function capacity. Campo et al. (2014) found that complaints of chronic cough significantly reduced the distance (51.76 m less; *p* = 0.04) that PLWH can walk during the 6MWT when compared to individuals who did not have this complaint.

Respiratory symptoms, such as breathlessness and complaints of cough, present more often in PLWH compared to HIV-negative individuals even when individuals are managed successfully on antiretroviral therapy (Brown et al. 2017). It therefore follows that monitoring and management of these symptoms during pulmonary rehabilitation interventions is of importance in this population as a means of influencing long-term health and wellness.

The lower level of physical function capacity observed in women when compared to men is expected in this cohort as it is in line with 6MWT findings reported by others (Britto et al. 2013; Casanova et al. 2011; Dourado, Vidotto & Guerra 2011; Mbada et al. 2013; Oliveira et al. 2018). Peak oxygen utilisation influences the distance that PLWH can achieve during the 6MWT (Oliveira et al. 2018; Oursler et al. 2009). Oliveira et al. (2018) reported that individuals with chronic lifestyle diseases, such as HIV, may not tolerate maximal exercise tests because of complaints of fatigue and pain, and results from such tests would thus not be because of reduced physical function, but more because of the symptoms experienced. Oliveira et al. (2018) thus propose that a sub-maximal exercise test, such as the 6MWT, would be more appropriate for use in such populations.

Oursler et al. (2009) reported that individuals with a lower VO_2_ peak tended to have moderate anaemia (haemoglobin 10–13 gm/dL). Anaemia is a common condition diagnosed in PLWH (Redig & Berliner 2013) and associated with a number of factors including female gender (Belperio & Rhew 2004).

Fatigue is a symptom associated with anaemia (World Health Organization 2017) and often reported by PLWH (Potterton 2016). It can negatively influence physical function and QOL of PLWH and when addressed improves these factors (Belperio & Rhew 2004). This could be why female participants in our study demonstrated a larger change in fatigue levels pre- to post-test. Change in breathlessness and fatigue levels did occur in this study cohort from pre- to post-test when performing the 6MWT. The change in breathlessness was significantly higher in female participants and a change of one unit rating higher on the Borg scale was noted. This finding is similar to results found by Casanova et al. (2011) where 50% of study participants reported an increase of breathlessness by greater than or equal to one unit on the Borg scale when performing the 6MWT.

The distance walked during the 6MWT in this cohort was higher than that reported by other authors in an African context. Mbada et al. (2013) assessed the health-related QOL and physical function capacity in a cohort of PLWH attending a virology research clinic in Ile-Ife, Nigeria. The authors found that male PLWH in their study walked farther (466.22 m; ± 89.9) than female participants (427.09 m; ± 94.32). When compared with non-infected HIV control participants (men: 600.09 m; ±112.59 and women: 526.07 m; ± 64.65), the difference was significantly lower (*p* = 0.001). Cobbing, Hanass-Hancock and Myezwa (2017) reported significantly lower 6MWT distance results in their population of PLWH in a peri-urban area in the eThekwini district in KwaZulu-Natal province of South Africa. Participants in their study walked 269.66 (± 74.55) m (intervention group) and 253.42 (± 79.79) m (control group) at baseline.

In their study, individuals were recruited for home-based rehabilitation if they had mobility limitations according to the World Health Organization (WHO) Disability Assessment Schedule. This could explain the shorter distances walked on the 6MWT for their cohort (Cobbing et al. 2017). Participants in our study were enrolled in a physical activity programme at the time of testing and few presented with comorbidities, which could explain the higher distances achieved on the 6MWT.

A number of 6MWT prediction reference equations are available for use in non-infected healthy individuals (Dourado et al. 2011). Factors known to predict individuals’ distance walked and that are included in such equations are age, gender, height, weight, BMI, resting heart rate and heart rate change (Britto et al. 2013; Burr et al. 2011; Casanova et al. 2011; Enright & Sherrill 1998; Gibbons et al. 2001). Even though a number of factors (age, gender, height, BMI, waist and hip circumferences) were associated with 6MWT distance findings in this cohort, only age and gender were included in the final model. The availability of a 6MWT distance prediction reference equation for PLWH in an African context could be of benefit to researchers and clinicians. A specific equation for an African context should be supported as differences in 6MWT findings in studies from different countries or regions are often not explained by differences in anthropometric measures, but more so because of geographical variations (Casanova et al. 2011) and other possible confounding variables, for example habits of study participants and nutritional status.

Home-based physical activity and rehabilitation programmes are used to manage and care for PLWH (Cobbing et al. 2017; Roos et al. 2014) and PTB survivors (De Grass, Manie & Amosum 2014) in an African context. During these programmes, walking is utilised to increase physical function capacity of individuals and capacity is usually assessed with the 6MWT.

The 6MWT prediction equation generated through this study is *y* = 699.50–80.95 (gender: women = 1; men = 0) – 2.35 (age). Our equation is similar to the equation proposed by Gibbons et al. (2001) for healthy individuals between the ages of 22 and 68 years, for example 868.8-(2.99 × age) – (74.7 × gender: women = 1; men = 0). Gibbons et al. (2001) included 79 participants in their study and their regression model accounted for 41% variability compared with 34% in our study. Further research is needed to validate the prediction equation produced in our study for the African region.

## Conclusion

Our study did not find that a previous medical history of PTB was a predictor of 6MWT distance achieved; however, several important associations were noted, which include age, gender, height, BMI, WC and hip circumference. This cohort only included 17 participants with a self-reported history of PTB. Further research regarding the association of a past medical history of PTB and distance walked during the 6MWT in PLWH is necessary with a larger study sample and possible age and gender matching to support our study findings. The inclusion of lung function test parameters would be of added benefit.
